# Identification and antimicrobial susceptibility of methicillin-resistant *Staphylococcus aureus*-associated subclinical mastitis isolated from dairy cows in Bogor, Indonesia

**DOI:** 10.14202/vetworld.2021.1180-1184

**Published:** 2021-05-13

**Authors:** Evi Nur Qolbaini, Miftahudin Majid Khoeri, Korrie Salsabila, Wisiva Tofriska Paramaiswari, Wisnu Tafroji, I. Made Artika, Dodi Safari

**Affiliations:** 1 Department of Biochemistry, IPB University, Jl. Raya Dramaga, Bogor, 16680, Indonesia; 2 Eijkman Institute for Molecular Biology, Jl. Diponegoro No. 69 Jakarta, 10340, Indonesia

**Keywords:** Bogor, dairy cows, methicillin-resistant *Staphylococcus aureus*, subclinical mastitis

## Abstract

**Background and Aim:**

Subclinical mastitis is an udder infection devoid of clinical symptoms, and *Staphylococcus aureus* is one of the bacteria causing this disease. This study aimed to identify and determine the prevalence and antibiotic susceptibility of methicillin-resistant *S. aureus* (MRSA)-associated subclinical mastitis isolated from dairy cows in Bogor, Indonesia.

**Materials and Methods:**

*S. aureus* was isolated from subclinical mastitis milk specimens. All strains were confirmed by polymerase chain reaction-based detection of staphylococcal *nuc*, and MRSA was confirmed by the presence of *mecA*. Antibiotic susceptibility was determined using the disk diffusion method.

**Results:**

From 86 milk samples, 49 isolates (57%) were confirmed as *S. aureus*. All *S. aureus* isolates were susceptible to tetracycline, gentamicin, chloramphenicol, erythromycin, and trimethoprim/sulfamethoxazole. Nine isolates were identified as MRSA (10.5%).

**Conclusion:**

In this study, we reported MRSA-associated subclinical mastitis in Bogor, Indonesia.

## Introduction

Globally, mastitis is an inflammation of the mammary gland, affecting dairy cows with high economic impact, including low milk production, increasing antibiotic residue contamination, and chronic infection, often leading to deaths [1,2]. It was reported that the prevalence of subclinical mastitis is higher than that of clinical mastitis [3]. Subclinical mastitis has a more common and serious impact on older lactating animals, contributing to most of the financial losses [2,4]. The major risk factors for developing mastitis include herd size, bedding material, and milking of mastitic cows [1,3]. Meanwhile, the age and number of parity (number of pregnancies carried by a cow) were significantly associated with the occurrence of subclinical mastitis [5]. At present, the diagnosis of subclinical mastitis is difficult and challenging to detect by visual inspection, and palpation of the udder than clinical mastitis due to the lack of any overt manifestation, such as visible changes in the udder or milk [6,7]. At present, several tests are being used to define mastitis, including physicochemical and biological diagnostics, for example, pH, electric conductivity, enzymes, biochemical molecules, and non-specific culture; somatic cell count; California mastitis test (CMT); digital mastitis detection tests; intramammary thermography; bio-sensors; and proteomic approaches[8].

*Staphylococcus* spp. (coagulase-negative) are the predominant contagious pathogens causing mastitis [3,9]. At present, *Staphylococcus aureus* infection of the mammary gland remains a major problem in the dairy industry worldwide [10]. Tarazona-Manrique *et al*. [11] reported that *Streptococcus agalactiae* and *S. aureus* were the most prevalent bacterial pathogens causing mastitis in specialized dairy herds in the Highlands of Boyacá, Colombia. It was also reported that *Staphylococcus* species were the dominant bacteria isolated from mastitis and subclinical mastitis cases in Ethiopia [1,5]. In Rwanda, among 123 crossbreed milking cows from 13 dairy farms, the prevalence of subclinical mastitis was mostly (50.4%) caused by the pathogens, coagulase-negative *Staphylococcus* (CNS), and *S. aureus* [4]. In Kenya, it was reported that the predominant bacteria from cow milk samples with clinical and subclinical mastitis were CNS followed by *Streptococcus* species, *S. aureus*, *Pseudomonas aeruginosa*, and *Enterobacter* species [3]. Methicillin-resistant *S. aureus* (MRSA) is a drug-resistant bacterial pathogen responsible for various infections worldwide, from humans to animals. MRSA strains have been detected in animals and are a frequent colonizer of animals, especially livestock, as new reservoirs. Livestock-associated MRSA (LA MRSA) causes infection in economically important livestock [12]. It was reported that 12.2% of mastitis cases were infected with MRSA isolated from milk samples obtained from the quarters [13]. For example, in Egypt, 35.7% of *S. aureus* strains isolated from milk samples of buffaloes and dairy cattle were cefoxitin resistant, and polymerase chain reaction (PCR) for *mecA* and *coa* genes revealed MRSA [14].

Recently, Ramandinianto *et al*. [15] reported that two MRSA strains were isolated from 150 cow milk samples from three village dairy farms in East Java, Indonesia. This study aimed to identify and determine the prevalence and antibiotic susceptibility of MRSA-associated subclinical mastitis isolated from dairy cows in Bogor, Indonesia.

## Materials and Methods

### Ethical approval

Ethical approval was not required for this study; however, samples were collected as per standard sample collection procedure.

### Study period and location

Milk samples were collected in 2013 and 2014 from cows with subclinical mastitis in a dairy cow farm in Bogor, Indonesia, as previously described [16].

### Milk sample collection

CMT-positive specimens (86/102) were used in this study [16]. One standard loop (0.01 mL) of the milk sample was streaked onto 5% sheep blood agar using the quadrant-streaking method for each sample. The plates were incubated aerobically at 37°C for 24-48 h. After incubation, colonies were identified according to their Gram reaction, cellular morphology, and catalase test [16]. The presumptive *S. aureus* isolates were stored at −80°C in a skim milk tryptone glucose glycerol medium [16].

### Molecular identification of *S. aureus* and MRSA

All isolates were subcultured on a blood agar plate (5%) and incubated at 37°C for 18-24 h. DNA was extracted using the heat shock method as previously described [17]. The harvested bacteria were placed into a 300 μL TE buffer, heated to 100°C for 5 min, and frozen at −20°C for 10 min. Then, they were centrifuged at 13,000× *g* for 10 min. The pellet and the supernatant were separated and stored at −30°C. All phenotypically identified isolates were confirmed using specific PCR that targeted the *thermonuclease* (*nuc*) gene with a forward primer (5’-TCAGCAAATGCATCACAAACAG-3’) and a reverse primer (5’-CGTAAATG CACTTGCTTCAGG-3’). Using PCR, the isolates resistant to cefoxitin or oxacillin were tested for the detection of the *mecA* gene. Specific primers for the *mecA* gene used reverse (5’-AACGATTGTGACACGATAGCC-3’) and ­forward (5’-GGGATCATAGCGTCATTATTC-3’) primers. For each reaction, a master mix (25 μL) containing GoTaq Green Master Mix (Promega, Madison, WI, USA), 25 μM primers 0.5 μL for each primer, nuclease-free water, and 2.5 μL DNA template was prepared. Conventional PCR was performed to detect the *mecA* gene under the following conditions: 5 min at 94°C; followed by 35 cycles of denaturation at 94°C for 30 s, annealing at 55°C for 30 s and extension at 72°C for 1 min, and a final extension at 72°C for 10 min [18,19].

### Antimicrobial susceptibility test

All *S. aureus* isolates were tested for antibiotic susceptibility using the disk diffusion method on Mueller-Hinton agar, according to the Clinical Laboratory Standards Institute (CLSI) 2019. The antimicrobial disks (Oxoid) contained the following: Chloramphenicol (30 µg), cefoxitin (30 µg), erythromycin (15 µg), sulfamethoxazole/trimethoprim (23.75/1.25 µg), gentamicin (10 µg), oxacillin (1 µg), and tetracycline (30 µg). Isolates resistant to cefoxitin and positive for the *mecA* gene were reported as MRSA, according to CLSI [20].

## Results

In this study, 73 presumptive *S. aureus* strains were isolated and identified from 86 milk samples with subclinical mastitis (84.5%). All strains were confirmed by PCR amplification of staphylococcal *nuc* (the PCR fragment length was 225 bp) and *mecA* genes (the PCR fragment length was 527 bp), resulting in 57.0% positivity for the nuc gene (49/86) and 10.5% positivity for *nuc* + *mecA* genes (9/86; MRSA) (Table-1).

**Table-1:** Identification of *S. aureus* and MRSA strains collected from 86 milk samples associated with subclinical mastitis from a dairy cow farm, Bogor, Indonesia.

*S. aureus* and MRSA identification	n (%)
Presumptive isolate	73 (84.9)
PCR positive for *nuc* gene	49 (57.0)
PCR positive for *nuc* and *mecA* genes (MRSA)	9 (10.5)

MRSA=Methicillin-resistant *S. aureus*, *S. aureus*= *Staphylococcus aureus*, PCR=Polymerase chain reaction

We used the disk diffusion method for the antibiogram. We found that all *S. aureus* strains were susceptible to tetracycline, gentamicin, chloramphenicol, erythromycin, and trimethoprim-sulfamethoxazole (Table-2). Meanwhile, 81.6% of the strains were susceptible to cefoxitin and oxacillin (Table-2). We found that all nine MRSA strains were non-susceptible to oxacillin and cefoxitin antibiotics. Here, the prevalence of MRSA among milk specimens with subclinical mastitis was 10.5% (9/86).

**Table-2:** Antibiogram profiles 49 *Staphylococcus aureus* strains collected from milk samples associated with subclinical mastitis from a dairy cow farm, Bogor, Indonesia.

Antimicrobial agent	Number (%) of susceptible strains
Gentamicin	49 (100)
Tetracycline	49 (100)
Erythromycin	49 (100)
Trimethoprim/sulfamethoxazole	49 (100)
Chloramphenicol	49 (100)
Cefoxitin	40 (81.6)
Oxacillin	40 (81.6)

## Discussion

MRSA is currently a global concern and has been reported as a major cause of health care-, community-, and livestock-associated infections [21,22]. MRSA has become an emerging and growing concern in companion and food-producing animals [23]. Our study found that 10.5% of MRSA strains were isolated and identified from milk specimens with subclinical mastitis from a dairy farm in Bogor, Indonesia. This finding agrees with the previous study of Guimarães *et al*. [13], who reported that 12.2% of MRSA was isolated from milk samples in São Paulo, Brazil. Recently, it was reported that the prevalence of *S. aureus* with PCR-positive mecA (MRSA) was 17.89% among milk and nasal swab samples from dairy cattle in Malaysia [24]. However, a lower rate (2%) of LA MRSA was observed from raw goat milk samples from the same country, Malaysia [25]. In Bangladesh, the prevalence of MRSA was 8.96% in clinical caprine mastitis cases [26].

Surveillance of MRSA prevalence causing mastitis and subclinical mastitis is important for the animals’ health because β-lactam drugs are still the most common antimicrobials used to treat mastitis, because alternative treatment with non-β-lactam antibiotics is limited [13,27]. Misuse and frequent use of antibiotics for prophylaxis or in animal feed to enhance growth play a crucial role in the emergence and spread of MRSA [28]. A majority of MRSA strains were more resistant to multiple antibiotics among *S. aureus* isolates recovered from bovine mastitis cases in Shanghai and Zhejiang areas of China [29]. In Bangladesh, ­multidrug-resistant *S. aureus* isolates from bovine mastitis milk samples were tetracycline, novobiocin, methicillin, vancomycin, and cephradine resistant [30]. Here, we found all MRSA strains with the *mecA* gene confirmed by the PCR method to be non-susceptible to oxacillin and cefoxitin. The presence of the *mecA* gene was also identified among oxacillin-susceptible *S. aureus* isolates [13].

### Limitation of the study

Our study was limited to only collecting milk samples from a dairy cow. In this study, we did not collect nasal swab samples from dairy workers. MRSA could be transmitted from animals, including dogs, cats, horses, small exotic animals, wildlife animals, and livestock, as a reservoir to humans, and vice versa [31,32]. The MRSA strains might be transmitted to humans by close contact and handling of dairy cows, as the same clonal complex of MRSA isolated from cow and dairy farmworkers was found [33]. MRSA is the most prevalent bacterium in conventional dairy farms, followed by organic, small, and large farms, in this order, and was correlated with lack of hygiene during milking [27]. The common route of MRSA transmission is through workers’ hands, udder cloths, and milking equipment, such as teat liners [27]. Good hygiene practices during milking, milk production, or animal handling; good husbandry; and biosecurity measures can reduce the risk of MRSA transmission in an animal population [21,27].

## Conclusion

Good hygiene practices during milking, milk production, or animal handling should be used to reduce the risk of MRSA transmission within a dairy farm. In summary, our study discovered 57% and 10.5% of *S. aureus* and MRSA, respectively, isolated from raw milk of subclinical mastitis dairy cows. This study demonstrated that antibiotics could be used to treat MRSA-associated subclinical mastitis in dairy cows.

## Authors’ Contributions

ENQ: Conception, design, analysis, and interpretation of data. MMK: Acquisition of data, and analysis and interpretation of data. KS: Acquisition of data, and analysis and drafted the manuscript. WTP: Acquisition of data, and analysis and interpretation of data. WT: Acquisition of data and revised the manuscript critically. IMA: Conception, design, and revised the manuscript critically. DS: Conception, design, interpretation of data, and drafted the manuscript. All authors have read and approved the final manuscript.
